# Tethered Magnets Are the Key to Magnetotaxis: Direct Observations of *Magnetospirillum magneticum* AMB-1 Show that MamK Distributes Magnetosome Organelles Equally to Daughter Cells

**DOI:** 10.1128/mBio.00679-17

**Published:** 2017-08-08

**Authors:** Azuma Taoka, Ayako Kiyokawa, Chika Uesugi, Yousuke Kikuchi, Zachery Oestreicher, Kaori Morii, Yukako Eguchi, Yoshihiro Fukumori

**Affiliations:** a School of Natural System, College of Science and Engineering, Kanazawa University, Kakuma-machi, Kanazawa, Japan; b Bio-AFM Frontier Research Center, College of Science and Engineering, Kanazawa University, Kakuma-machi, Kanazawa, Japan; c Department of Life Science, Graduate School of Natural Science and Technology, Kanazawa University, Kakuma-machi, Kanazawa, Japan; d School of Environment and Natural Resources, The Ohio State University, Columbus, Ohio, USA; University of California, Berkeley; California Institute of Technology/HHMI

**Keywords:** actin-like protein, bacterial organelle, cytoskeleton, live-cell imaging, magneto-reception, magnetosome

## Abstract

Magnetotactic bacteria are a unique group of bacteria that synthesize a magnetic organelle termed the magnetosome, which they use to assist with their magnetic navigation in a specific type of bacterial motility called magneto-aerotaxis. Cytoskeletal filaments consisting of the actin-like protein MamK are associated with the magnetosome chain. Previously, the function of MamK was thought to be in positioning magnetosome organelles; this was proposed based on observations via electron microscopy still images. Here, we conducted live-cell time-lapse fluorescence imaging analyses employing highly inclined and laminated optical sheet microscopy, and these methods enabled us to visualize detailed dynamic movement of magnetosomes in growing cells during the entire cell cycle with high-temporal resolution and a high signal/noise ratio. We found that the MamK cytoskeleton anchors magnetosomes through a mechanism that requires MamK-ATPase activity throughout the cell cycle to prevent simple diffusion of magnetosomes within the cell. We concluded that the static chain-like arrangement of the magnetosomes is required to precisely and consistently segregate the magnetosomes to daughter cells. Thus, the daughter cells inherit a functional magnetic sensor that mediates magneto-reception.

## IMPORTANCE

Half a century ago, bacterial cells were considered a simple “bag of enzymes”; only recently have they been shown to comprise ordered complexes of macromolecular structures, such as bacterial organelles and cytoskeletons, similar to their eukaryotic counterparts. In eukaryotic cells, the positioning of organelles is regulated by cytoskeletal elements. However, the role of cytoskeletal elements in the positioning of bacterial organelles, such as magnetosomes, remains unclear. Magnetosomes are associated with cytoskeletal filaments that consist of the actin-like protein MamK. In this study, we focused on how the MamK cytoskeleton regulates the dynamic movement of magnetosome organelles in living magnetotactic bacterial cells. Here, we used fluorescence imaging to visualize the dynamics of magnetosomes throughout the cell cycle in living magnetotactic bacterial cells to understand how they use the actin-like cytoskeleton to maintain and to make functional their nano-sized magnetic organelles.

## INTRODUCTION

Magnetotactic bacteria (MTB) rely on magnetic organelles called magnetosomes to perform magneto-aerotaxis, which strongly enhances their capacity to orient along the geomagnetic field to find a favorable microaerobic habitat (1–4). MTB typically have many magnetosomes that form a long chain running the length of the interior of the cell. The actin-like cytoskeletal protein MamK mediates the alignment of magnetosomes (5, 6); however, the detailed mechanism and physiological significance of the chain-like structure of magnetosomes and its positioning have been poorly understood. Bacterial actin-like proteins are diverse and perform specific functions, such as mediating DNA segregation, maintaining cell shape, and participating in cell division (7, 8). MamK is an evolutionarily conserved actin-like protein produced by MTB that forms cytoskeletal filaments that associate with magnetosome chains (5, 6). MamK is the sole bacterial actin-like protein associated with organelle organization (7, 8). Studies of MamK that have yielded insights into its function have mainly relied on *in vitro* biochemical examinations (9–12) or acquisitoin of static (nondynamic) images using electron microscopy (5, 13). Studies using cryo-electron tomography have shown that the structure of the magnetosome chain is disorganized in *mamK* deletion mutants of *Magnetospirillum magneticum* AMB-1 (5) and *Magnetospirillum gryphiswaldense* MSR-1 (13), indicating that the MamK cytoskeleton mediates the formation and organization of the magnetosome chain. However, these studies were based on observations of static electron microscopic images (5, 13). Although the dynamics of eukaryotic organelles and cytoskeletons have been extensively studied, relatively few studies have focused on bacteria (14, 15). For example, the role of MamK in magnetosome segregation was studied using time-lapse live-cell imaging of the model magnetotactic bacterium *M. gryphiswaldense* MSR-1 (15). Those authors revealed that magnetosome chains are segregated by dynamic repositioning from the cell pole to the midcell of daughter cells during cytokinesis, suggesting that magnetosome motion depends on the treadmill action of MamK filaments.

Here, we used *M. magneticum* AMB-1 (AMB-1), which is similar to MSR-1 but serves as another model of magnetotactic bacteria, to visualize the dynamics of magnetosomes in living cells and to identify the function of the MamK cytoskeleton during magnetosome segregation. We developed a live-cell time-lapse fluorescence imaging technique to analyze the subcellular dynamics of magnetosomes in AMB-1 cells. We used highly inclined and laminated optical sheet (HILO) microscopy (16) to generate images with a high signal-to-noise ratio to observe the dynamics of magnetosomes during the entire cell cycle of AMB-1 cells. We showed that MamK is required to prevent the intracellular diffusion of magnetosomes that allows them to segregate equally the magnetosomes to the daughter cells and function as a stable magnetic sensor. We found that MamK is required to maintain the organization of magnetosomes and that MamK ATPase activity is required for its function.

## RESULTS

### Visualization of the dynamics of magnetosomes throughout the cell cycle via HILO microscopy.

To visualize the dynamics of magnetosomes in living cells, green fluorescent protein (GFP) was fused to the magnetosome membrane proteins MamI and MamC and expressed in AMB-1 cells. MamI, which is essential for the formation of magnetosome membrane vesicles (17), can be used to detect vesicles with and without magnetite (18). MamC regulates the size and shape of magnetite crystals in magnetosomes. Immunoblot analyses showed that both MamI-GFP and MamC-GFP localized in the magnetosomes (see Fig. S1A in the supplemental material), although their localization patterns differed (Fig. S1B and C). MamI-GFP was organized into a linear, continuous chain (Fig. S1B) which was described previously (18), while MamC-GFP formed a patchy chain (Fig. S1C) that had the same localization pattern as magnetite-bearing magnetosomes (Fig. S1D). Therefore, it is feasible that the mineralizing protein MamC can be used as an indirect means to specifically detect the positions of mineral-containing magnetosomes. The expression of the GFP-fusion proteins did not affect magnetite growth or magnetization (Table S1). We estimated the protein contents of each subcellular fraction: magnetosome, membrane, and soluble fractions (see Materials and Methods). The magnetosome fraction contained ~0.1% cellular proteins. According to the immunoblotting band intensities and the ratio of protein contents in each fraction, ~40% of MamC-GFP and ~3% of MamI-GFP localized in the magnetosome fractions, confirming the specific localization of both GFP-tagged proteins (Fig. S1E). The MamI-GFP content in the magnetosome fraction may have been an underestimate, because a portion of the MamI-labeled empty magnetosome vesicles was lost to the cell membrane fraction during the magnetic purification process. The fluorescence intensity of MamI-GFP was lower than that of MamC-GFP and decreased during the 24 h of time-lapse image acquisition. Therefore, in order to visualize magnetosomes for the entire cell cycle, we used MamC-GFP for longer time-lapse observations.

10.1128/mBio.00679-17.1 FIG S1  (A) Localization of MamI-GFP and MamC-GFP in wild-type and Δ*mamK M. magneticum* AMB-1 cells. Immunoblotting results with anti-GFP antibody of proteins (10 µg/lane) extracted from the soluble, membrane, and magnetosome fractions are shown. Both GFP-fused MamI and MamC were predominantly located in the magnetosome fractions. (B and C) Subcellular localization of MamI-GFP and MamC-GFP. Merged GFP and bright field images of cells expressing MamI-GFP (B) and MamC-GFP (C). (D) Transmission electron microscope image of *M. magneticum* AMB-1 cells. The red circles show the positions of empty vesicles. (E) Summary table of MamI-GFP and MamC-GFP localization, estimated from immunoblotting. Band intensities were measured in immunoblots of the soluble, membrane, and magnetosome fractions (5 µg protein/lane) prepared from wild-type cells. Contents of MamI-GFP and MamC-GFP in each fraction were calculated using the ratio of immunoblotting band intensities and the percentages of protein contents of each subcellular fraction. Download FIG S1, PDF file, 1.5 MB. Copyright © 2017 Taoka et al. 2017 Taoka et al. This content is distributed under the terms of the Creative Commons Attribution 4.0 International license.

10.1128/mBio.00679-17.7 TABLE S1  Phenotypes of strains. Download TABLE S1, DOC file, 0.03 MB. Copyright © 2017 Taoka et al. 2017 Taoka et al. This content is distributed under the terms of the Creative Commons Attribution 4.0 International license.

**FIG 1:**
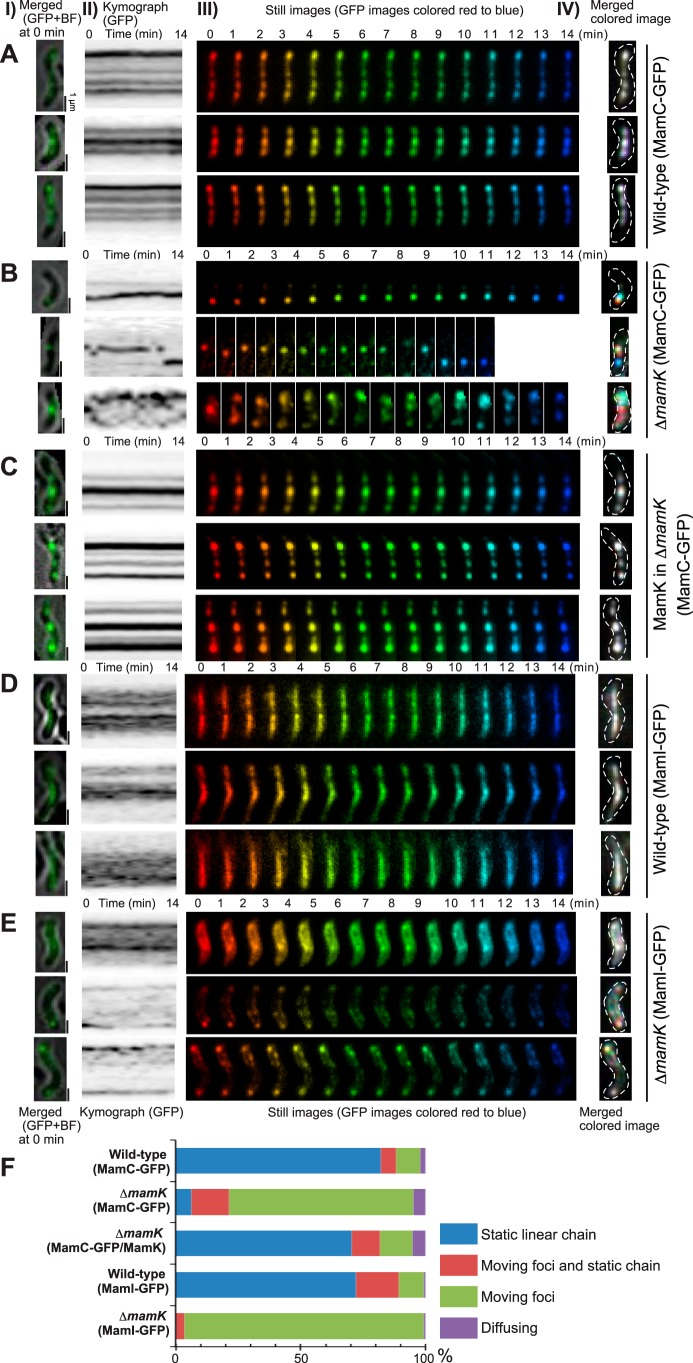
Dynamics of magnetosomes. Magnetosomes were visualized using MamC-GFP (A to C) and MamI-GFP (D and E) in wild-type (A and D), Δ*mamK* deletion mutant (B and E), and MamK-complemented Δ*mamK* deletion mutant (C) cells, respectively. (Column I) Merged GFP and bright-field images of cells at time zero. Scale bars, 1 µm. (Column II) Kymographs of the GFP trajectory in maximum projections. (Column III) Time-lapse still images acquired during a 14-min interval are sequentially rainbow colored, red to blue. (Column IV) Merged images of the rainbow-colored still images in column III. White indicates the static GFP signal, and colored signals show the dynamic GFP signal. The images show that MamK is dynamic in Δ*mamK* deletion mutant cells and static in wild-type and MamK-complemented cells over a time scale of minutes. (F) Dynamic localization patterns of magnetosomes. The dynamics of magnetosomes were determined based on time-lapse observation for 3 h in MamC-GFP-expressing wild-type cells (*n* = 107) and in Δ*mamK* mutant cells (*n* = 122) and in MamI-GFP-expressing wild-type cells (*n* = 141) and in Δ*mamK* mutant cells (*n* = 145). For the complementation experiment, wild-type MamK was coexpressed with MamC-GFP in deletion mutant Δ*mamK* cells (*n* = 192). In Δ*mamK* mutant cells, magnetosomes labeled with both MamC-GFP and MamI-GFP were mostly dynamic, whereas wild-type and MamK-complemented cells had mostly static magnetosomes.

**FIG 2:**
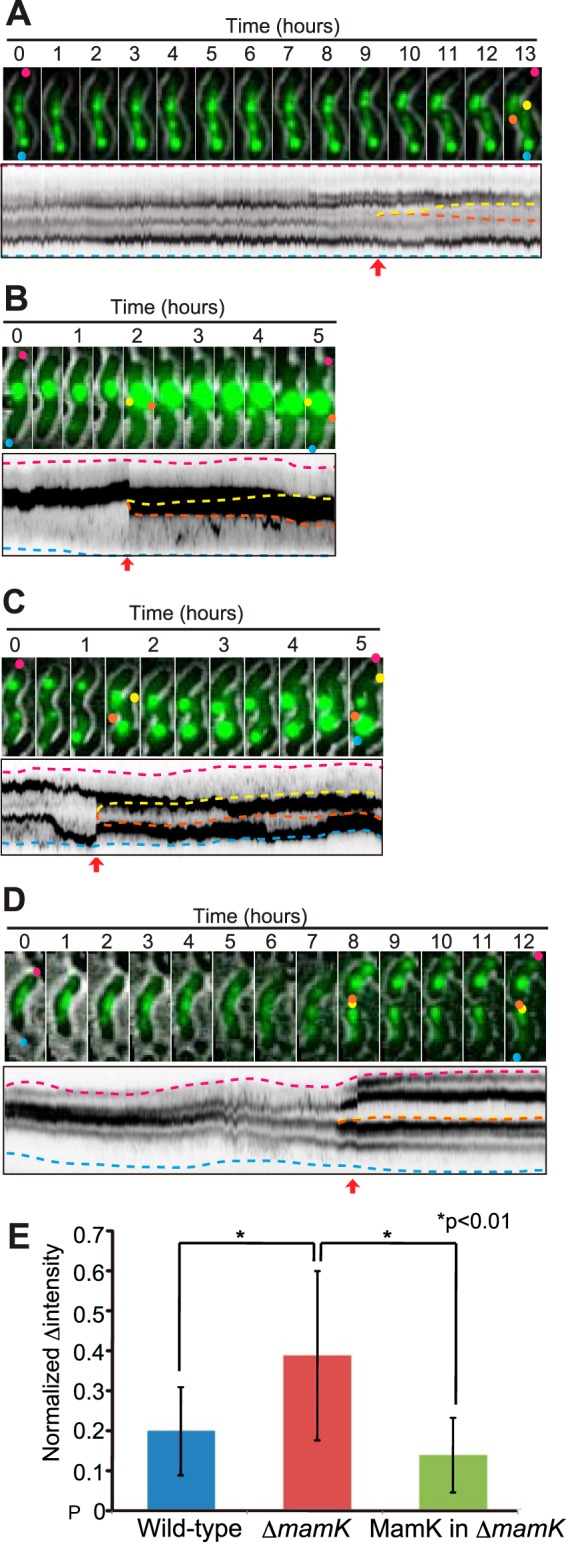
Segregation of magnetosomes to daughter cells. Time-lapse still images and kymographs in maximum projection of magnetosomes in wild-type (A), Δ*mamK* deletion mutant (B and C), and MamK-complemented Δ*mamK* deletion mutant (D) cells. The pink and blue dashed lines in the kymographs show the positions of parental cell poles, and the orange and yellow dashed lines show the newly synthesized poles of daughter cells. The red arrows show the times of cell division. (E) Observed errors of magnetosome segregation with the standard deviation (*n* = 20). The normalized changes (Δ) in intensities were calculated from the following equation:$\sqrt{[(A-B)/(A+B){]}^{2}}$, where *A* and *B* represent the fluorescence intensities of individual daughter cells derived from the parental cell. The small, normalized changes in intensities indicate precise segregation. The differences between populations were evaluated using a *t* test (*P* < 0.01). These results show that static magnetosomes are critical for proper segregation of magnetosomes to daughter cells.

The fluorescent labeling of magnetosomes visualized using HILO microscopy did not significantly affect the doubling times of cells (6 to 10 h) compared to labeled cells grown in batch cultures (approximately 6 h), indicating that the imaging procedure had no effect on the cells (Table S1; Movie S1). According to the live-cell imaging results, the magnetosomes were fixed in a stable chain-like arrangement in the wild-type cells. As shown in Fig. 1, the time-lapse still images of the MamC-GFP and MamI-GFP signals were sequentially rainbow colored from red to blue for the entire 14-min observation. The colored images were superimposed to create a single-color image of all of the different colored time-lapse still images. The merged colored image shows how static or dynamic the labeled proteins are during the time-lapse observation. Thus, the white and colored spots indicate static and dynamic GFP signals, respectively. The magnetosomes in wild-type cells were white during the 14-min observation (Fig. 1A and D), indicating static localization along the long axis of the cell. Movie S2 shows a 17-h-long time-lapse observation of magnetosomes from wild-type cells. The static localization of magnetosomes was maintained during the entire cell cycle (cell growth, cell division, and post-cell division), as indicated by the parallel lines in the kymographs of GFP fluorescence (Fig. 2A; Movie S3).

10.1128/mBio.00679-17.3 MOVIE S1  A time-lapse movie showing the growth of cells under imaging conditions. Frames are 1 min apart. Time (hours and minutes) is indicated on the top left. Download MOVIE S1, MOV file, 12.5 MB. Copyright © 2017 Taoka et al. 2017 Taoka et al. This content is distributed under the terms of the Creative Commons Attribution 4.0 International license.

10.1128/mBio.00679-17.4 MOVIE S2  Representative time-lapse movie of wild-type cells expressing MamC-GFP. Frames are 1 min apart. Time (hours and minutes) is indicated on the top left. Download MOVIE S2, AVI file, 9.2 MB. Copyright © 2017 Taoka et al. 2017 Taoka et al. This content is distributed under the terms of the Creative Commons Attribution 4.0 International license.

10.1128/mBio.00679-17.5 MOVIE S3  The time-lapse movie of the cells for which results are shown in Fig. 2A, B, and C for the 13-h observation, 5-h observation, and 5-h observation, respectively. Frames are 1 min apart. Download MOVIE S3, MOV file, 8 MB. Copyright © 2017 Taoka et al. 2017 Taoka et al. This content is distributed under the terms of the Creative Commons Attribution 4.0 International license.

### The MamK cytoskeleton anchors magnetosomes to prevent their simple diffusion.

The merged colored time-lapse images of mutant Δ*mamK* cells indicated that the magnetosomes were very dynamic during the 14 min of observation (Fig. 1B and E). MamC- and MamI-labeled magnetosomes in Δ*mamK* mutant cells were detected as multicolored spots, indicating that the magnetosomes traveled around the cells on a timescale of minutes. The magnetosomes randomly moved throughout the cell, forming small, fast-moving fluorescent foci or large slow-moving fluorescent foci during both the 14-min (Fig. 1B and E) and longer (Fig. 2B and C) time-lapse imaging, in contrast to the static straight chain observed in wild-type cells (Fig. 1A and D and 2A). Figure 1F illustrates the different patterns of magnetosome dynamics among wild-type, mutant Δ*mamK*, and MamK-complemented cells based on a 3-h time-lapse observation. Static chain-like magnetosomes were observed in 70 to 80% of wild-type cells, whereas in the mutant Δ*mamK* cells approximately 70% contained moving magnetosomes that were scattered throughout the cell and 6% had static chain-like structures (Fig. 1F). According to the 24-h time-lapse images, the small foci of magnetosomes moved around within the cells while the larger bright foci maintained their positions or moved slowly (Movie S4). When mutant Δ*mamK* cells were transformed with a plasmid that expressed MamK, the static chain-like positioning of magnetosomes was restored (Fig. 1F). The recovery of static magnetosome localization in the MamK-complemented cells was observed in both the 14-min (Fig. 1C) and longer (Fig. 2D) time-lapse imaging. The expression level and localization of MamK in the complemented cells was similar to those of wild-type cells (Fig. S2).

10.1128/mBio.00679-17.2 FIG S2  Expression of MamK^WT^, MamK^E143A^, and MamK^D161A^ in Δ*mamK* mutant cells. (A) Immunoblotting with anti-MamK antibody of cell-free extracts (10 µg protein/lane) from wild-type, Δ*mamK* deletion mutant, and MamK-complemented strains. The MamK content of the complemented strain was similar to that of the wild type. (B) Immunoblotting with the anti-MamK antibody of cell-free extracts (10 µg protein/lane) from the MamK-complemented strain and from MamK^E143A^ and MamK^D161A^ expressed in Δ*mamK* mutant cells. (C) Immunofluorescence images of wild-type cells (column I), MamK-complemented cells (column II), MamK^E143A^-expressing cells (column III), and MamK^D161A^-expressing Δ*mamK* deletion mutant cells (column IV) with the anti-MamK antibody. The MamK ATPase mutants MamK^E143A^ and MamK^D161A^ showed linear filamentous localization similar to MamK^WT^. Download FIG S2, PDF file, 0.8 MB. Copyright © 2017 Taoka et al. 2017 Taoka et al. This content is distributed under the terms of the Creative Commons Attribution 4.0 International license.

10.1128/mBio.00679-17.6 MOVIE S4  Representative time-lapse movie of *mamK* deletion mutant cells expressing MamC-GFP. Frames are 1 min apart. Time (hours and minutes) is indicated on the top left. Download MOVIE S4, AVI file, 17.9 MB. Copyright © 2017 Taoka et al. 2017 Taoka et al. This content is distributed under the terms of the Creative Commons Attribution 4.0 International license.

**FIG 3:**
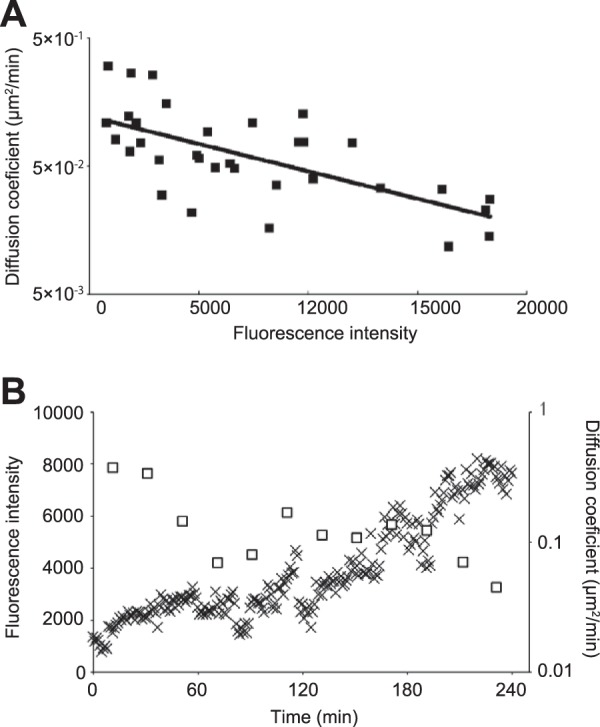
Relationship between diffusion coefficients and fluorescence intensities of MamC-GFP foci in Δ*mamK* deletion mutant cells. (A) The diffusion coefficient was calculated from the slope of the MSD versus time lag plot (*n* = 33). The MSD was determined using the trajectory of the center positions of MamC-GFP foci in 10 mutant Δ*mamK* cells. The solid line represents an exponential curve fitted to the data points (*R*^2^ = 0.44). (B) The time course of the fluorescence intensities and diffusion coefficients, calculated from a single focus in a Δ*mamK* mutant cell. The open squares show the diffusion coefficient, and the cross marks show the fluorescence intensity. The diffusion coefficients were calculated by measuring the trajectories of the center position of the focus over the course of 20 frames.

In mutant Δ*mamK* cells, the movement of magnetosomes was less than 1 µm during each 1-min time interval (Fig. 1B and E). We calculated diffusion coefficients and measured fluorescence intensities of individual MamC-GFP fluorescent foci in Δ*mamK* strain cells (Fig. 3A). The diffusion coefficients of 33 randomly selected foci in 10 mutant Δ*mamK* cells were calculated using 20 to 100 frames (1 frame/min) from the time-lapse images. The relationship between the diffusion coefficients and the florescent intensities of the MamC-GFP foci showed a negative correlation (Fig. 3A). The diffusion coefficient of the focus possessing the lowest fluoresce intensity was approximately 0.1 µm^2^/min, while that of the focus possessing the highest fluoresce intensity was approximately 0.02 µm^2^/min. Moreover, we occasionally found that a focus in Δ*mamK* mutant cells gradually strengthened its fluorescence intensity over time and, simultaneously, the diffusion coefficient decreased (Fig. 3B). A lower diffusion coefficient value means slower movement. As the fluorescent intensity of MamC-GFP increased, the movement of individual magnetosomes slowed down, and generally, the diffusion coefficient of an object was negatively correlated with its molecular size. Thus, in Fig. 3B, it is likely that the increase of the fluorescence intensity of the focus over time indicates a lengthening of the magnetosome chain via accrual of magnetosomes.

Kalwarczyk et al. determined a formula for the relationship between diffusion coefficients and sizes of simple diffusing macromolecules, such as proteins, mRNA, plasmids, and 70S ribosomes, in *Escherichia coli* cells (19). We used this formula to predict the diffusion coefficient for a single magnetosome by simple diffusion to be ~0.4 µm^2^/min and that of a chain of 10 to 30 magnetosomes to range from 0.03 to 0.01 µm^2^/min (see Materials and Methods for the calculations). The observed diffusion coefficients of the MamC-GFP foci ranged from 0.1 to 0.02 µm^2^/min (from the fit curve in Fig. 3A). In mutant Δ*mamK* cells, the magnetosomes moved by simple diffusion in the absence of anchoring molecules, as indicated by how well the values of observed magnetosome foci movements fit to the calculated values of simple diffusion. Therefore, we conclude that the purpose of MamK is to anchor magnetosomes in a chain to prevent their simple diffusion.

### The static chain-like arrangement is required for precise segregation of magnetosomes.

Time-lapse imaging revealed that magnetosomes in mutant Δ*mamK* cells were propagated unevenly to daughter cells during cell division (Fig. 2B and C; Movie S3). Figure 2E shows the differences in segregation patterns of magnetosomes between wild-type, mutant Δ*mamK*, and the MamK-complemented strains. The normalized differences in GFP fluorescence intensities between mutant Δ*mamK* daughter cells were significantly higher than in wild-type and MamK-complemented cells (Fig. 2E), indicating that stationary magnetosomes were required for their equal segregation to daughter cells. These findings are consistent with those of a previous report which found that the number of magnetosomes containing magnetite decreases during exponential growth of mutant Δ*mamK* versus wild-type cells (20).

### MamK ATPase activity is required for static magnetosome positioning.

**FIG 4:**
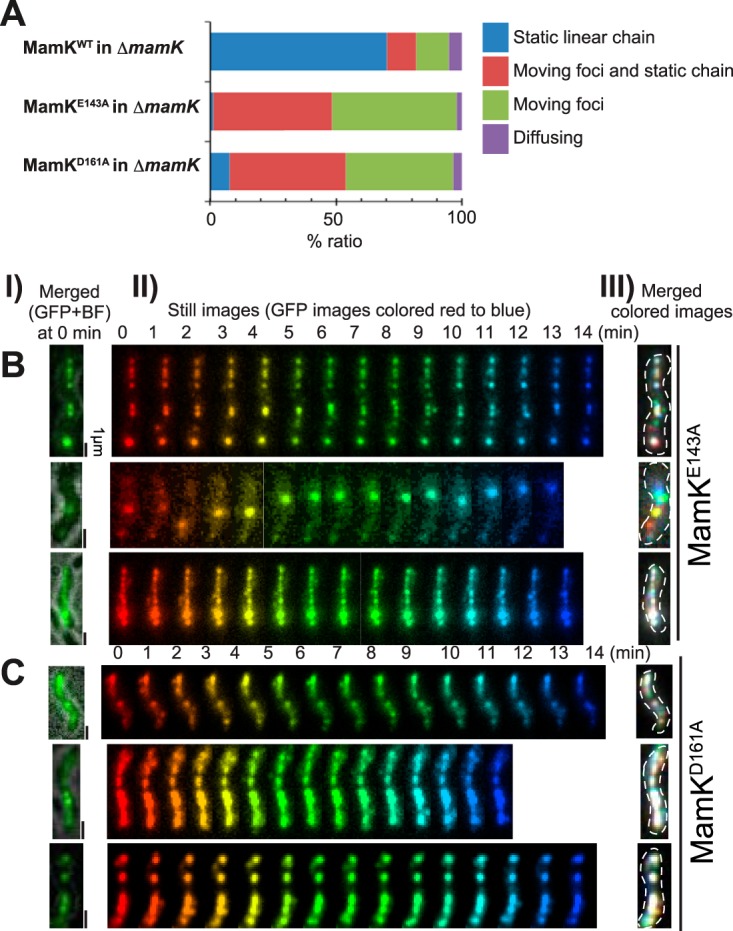
MamK ATPase activity is required for anchoring magnetosomes. (A) Dynamic localization patterns of magnetosomes. The dynamics of magnetosomes were determined using time-lapse imaging for 3 h in Δ*mamK* mutant cells expressing wild-type (WT) MamK (*n* = 192), MamK^E143A^ (*n* = 151), or MamK^D161A^ (*n* = 380), respectively, with MamC-GFP. (B and C) Effects of expression of ATPase-defective MamK, (B) MamK^E143A^, and (C) MamK^D161A^ on the dynamics of magnetosomes. (Column I) Merged GFP and bright-field images of the cells at time zero. (Column II) Time-lapse images acquired for 14 min at 1-min intervals and sequentially rainbow colored from red to blue. (Column III) Merged images of the rainbow-colored still images in (II). Note that a single cell expressing MamK^E143A^ and MamK^D161A^ contains static (white) and dynamic (colored) magnetosomes. Moreover, the dynamic magnetosomes continuously attached and detached to the long axis of the cell, suggesting that magnetosomes were loosely attached to the ATPase-defective MamK filaments.

The ATPase active site of MamK is conserved among actin-like proteins, and MamK-ATPase activity is not required for polymerization into filaments (11), although it is required to form dynamic filaments in cells (21). Previous work showed that MamK^E143A^ and MamK^D161A^ lacked ATPase activity and lost filament dynamics *in vivo* (15). We used MamK^E143A^ and MamK^D161A^ to investigate the function of MamK-ATPase in magnetosome anchoring (Fig. 4). The localization and expression levels of MamK^E143A^ and MamK^D161A^ were similar to those of wild-type MamK (Fig. S2B and C). However, ATPase-defective MamK mutants could not rescue the stable linear magnetosome positioning within the cell (Fig. 4A). MamK^E143A^ and MamK^D161A^ cells showed an intermediate phenotype of magnetosome dynamics compared with those of the wild-type and mutant Δ*mamK* cells. The merged colored images (Fig. 4B and C) showed that cells contained both static (white) and dynamic (colored) magnetosomes. In mutant Δ*mamK* cells, magnetosomes were dispersed throughout the cell; in contrast, the dynamic, colored magnetosomes localized along the long axis of MamK^E143A^ and MamK^D161A^ cells. These results suggest that the magnetosomes were not securely attached to the ATPase-defective MamK filaments, indicating that MamK ATPase activity was required for capturing all of the magnetosomes on the MamK cytoskeletal filaments.

## DISCUSSION

The magnetosome is a distinct, membrane-enclosed structure that localizes specialized proteins, associates with cytoskeletal filaments, and contributes to a specific function, properties that are very similar for eukaryotic organelles. Thus, the magnetosome serves as a model of a bacterial organelle. However, little is known about the dynamic nature of bacterial organelles within living cells because of their small size, making direct imaging via light microscopy difficult. Here, we visualized the dynamics of magnetosome organelles in live cells throughout the cell cycle by using the high temporal-spatial resolution afforded by fluorescence imaging using HILO microscopy. We acquired over 1,400 time-lapse HILO images to produce a time-lapse video of magnetosome dynamics in growing cells over the course of 24 h at 1-min time intervals. This high temporal resolution gives our live-cell magnetosome videos a distinct advantage over previous work that used live-cell time-lapse fluorescence imaging of magnetosomes (15, 22).

Previous work using cryo-electron microscopy (cryo-EM) observations repeatedly demonstrated that magnetosomes in a mutant Δ*mamK* cell had a chain-like configuration (5, 13, 23). Whereas, cryo-EM gives a snapshot of the near-native intracellular structure with nano-order spatial resolution, HILO microscopy does not have enough spatial resolution to demonstrate whether the magnetosomes form a chain or an aggregate in a cell. In our study, live-cell imaging of magnetosomes in wild-type and mutant Δ*mamK* cells revealed that the MamK cytoskeleton is essential for maintaining magnetosomes in a static chain-like arrangement throughout the cell cycle (Fig. 1). The magnetosomes in mutant Δ*mamK* cells underwent random dynamic motion or formed a large off-center fluorescent focus in the cell. Based on a combination of the cryo-EM images and our HILO results, the magnetosome chain assembled in a MamK-independent manner and diffused dynamically in mutant Δ*mamK* cells.

Katzmann et al. performed time-lapse microscopy of mutant Δ*mamK M. gryphiswaldense* MSR-1 cells to image magnetosome chain assembly after iron induction (13). They showed that the magnetosomes in mutant Δ*mamK* cells are maintained in a chain; however, they were fragmented and displaced in a manner similar to the localization in our study. On the other hand, Cornejo et al. genetically generated an inducible magnetosome-forming strain of *M. magneticum* AMB-1 and observed magnetosome formation using cryo-EM (23). They suggested that there are two distinct phases to magnetosome chain assembly, a long-range mechanism and a short-range mechanism. In the long-range mechanism, individual magnetosomes are discontinuously aligned along the length of the cell and are connected by a short-range mechanism using the MamK cytoskeleton. Our HILO results suggest that the magnetosome chain is arranged by the long-range mechanism, which cannot rigidly fix the magnetosome chain and does not keep the individual magnetosomes in a given position at midcell.

As the time-lapse study progressed, the MamC-GFP fluorescence intensity increased, indicative of the magnetosome chain increasing in size, which coincided with the diffusion coefficient decreasing (Fig. 3B), meaning that there was less magnetosome movement. In addition, as shown in Fig. 3A, the distance that magnetosomes traveled in mutant Δ*mamK* cells suggested that magnetosomes move through simple diffusion, as do other subcellular macromolecules, such as ribosomes and plasmids. Therefore, the MamK cytoskeleton anchors magnetosomes in the cell. In a previous study, cryo-electron tomographic images of AMB-1 cells showed that magnetosome vesicles were derived from the invagination of the inner membrane and were always connected to it by a narrow channel (5). Recently, the diffusion coefficients of glycosylphosphatidyl inositol-anchored large proteins in the plasma membrane of trypanosomes have been estimated by using fluorescence recovery after photobleaching analysis of live cells (24). According to this study, the diffusion coefficients of the membrane-integrated proteins, which ranged from 3 to 20 nm in diameter, were 0.6 to 2.6 µm^2^/min. These values were on the same order as that of MamC-GFP-labeled magnetosomes observed in our study (Fig. 3A). Together, our results indicate that the narrow channel connecting the magnetosome vesicle and the inner membrane does not contribute to fixing the location of magnetosomes. Therefore, the magnetosomes in the mutant Δ*mamK* cells probably diffuse on the peripheral surface of the inner membrane.

According to the immunoblotting analysis of the wild-type subcellular fractions, 40% of MamC-GFP was concentrated in the magnetosomes (Fig. S1E), suggesting that the GFP signal in the fluorescence microscopic images exhibits magnetosome localization. This estimation assumes that the other 60% of the MamC-GFP signal is homogeneously localized throughout the cell body, and therefore could have altered its localization, e.g., forming aggregates outside magnetosomes, in the cytoplasm. However, in wild-type cells, we did not observe any indication of this, and we mostly saw a linear chain of fluorescence foci; therefore, there is no reason to assume that the excess MamC-GFP signal would cause foci in the cytoplasm. In the mutant Δ*mamK* cells, we found nonlinear foci throughout cells, and we assume that these were in fact magnetosomes and not random clusters of MamC-GFP signals.

Toro-Nahuelpanet al. used a similar live-cell imaging technique to observe MSR-1 cells to show the repositioning of the magnetosome chain (15). The magnetosome chain of MSR-1 cells undergoes repositioning from the new pole toward the middle of the daughter cell before cytokinesis is completed during the cell division process. The midcell localization of magnetosomes as a single chain in living MSR-1 cells was observed by *in vivo* time-lapse fluorescence imaging using MamC-GFP (15). In contrast, we found that the position of the magnetosome chain in AMB-1 cells was kept static before and after cytokinesis, and repositioning of magnetosome chains was not observed. These two different observations indicate that the mechanism of magnetosome segregation is species specific. The differences in segregation mechanisms may be explained by the differences in magnetosome structures between MSR-1 and AMB-1 cells. Although both species have a magnetosome chain along the long axis of their cells, a single chain of magnetite-bearing magnetosomes was observed in the middle of MSR-1 cells, in contrast to the separated multiple chains of magnetite-bearing magnetosomes in AMB-1 cells (Fig. S1D). These observations suggest that dynamic repositioning of the magnetosome chain during cytokinesis achieves a midcell localization as a single chain in MSR-1 cells versus its static positioning as multiple chains along the cell body in AMB-1 cells.

The results of the live-cell imaging of *M. magneticum* AMB-1 showes that the MamK cytoskeleton tethers magnetosomes in a static chain to prevent diffusion or aggregation of magnetosomes by a physical disturbance, such as simple diffusion. The static chain-like magnetosome arrangement is required to precisely segregate the magnetosomes to daughter cells. Thus, the daughter cells will inherit a functional magnetic sensor that ensures magneto-aerotaxis will be propagated to the next generation of cells. This function of MamK is likely accomplished through the treadmill dynamics of MamK filaments, with MamK ATPase activity necessary for the dynamics of the MamK filaments (15, 21). Now that we have established how MamK functions in living cells, the next step will be to determine the mechanism of capturing and anchoring magnetosome by MamK filaments. Our newly developed method allows us to visualize the dynamic nature of nano-sized bacterial organelles by recording time-lapse images of living cells during the cell cycle. This technique will facilitate future research into the function of proteins exclusive to magnetotactic bacteria as well as their unique bacterial organelles.

## MATERIALS AND METHODS

### Bacterial strains, cultures, plasmids, and primers.

*M. magneticum* AMB-1 and *E. coli* strains used in this study are listed in Table S2. *M. magneticum* AMB-1 strains were cultured in a chemically defined liquid medium (MS-1 medium) at 28°C in the dark (25). *E. coli* strains were cultivated in LB broth (26) at 37°C, unless specified otherwise. When necessary, antibiotics were added at the following concentrations: for AMB-1, kanamycin at 5 µg/ml; for *E. coli*, kanamycin at 20 µg/ml and trimethoprim at 100 µg/ml. Plasmids and primers are listed in Tables S2 and S3, respectively.

10.1128/mBio.00679-17.8 TABLE S2  Bacterial strains and plasmids used in this study. Download TABLE S2, DOC file, 0.04 MB. Copyright © 2017 Taoka et al. 2017 Taoka et al. This content is distributed under the terms of the Creative Commons Attribution 4.0 International license.

10.1128/mBio.00679-17.9 TABLE S3  Primers used in this study. Download TABLE S3, DOC file, 0.04 MB. Copyright © 2017 Taoka et al. 2017 Taoka et al. This content is distributed under the terms of the Creative Commons Attribution 4.0 International license.

### Plasmid construction.

To express recombinant proteins in AMB-1 cells, the broad-host-range protein expression vector pBBR111 harboring the isopropyl-β-d-thiogalactopyranoside (IPTG)-inducible *tac* promoter (27) was used. An infusion cloning system (TaKaRa Bio) was used for the cloning procedures. The DNA fragment encoding *gfp* was PCR amplified using the template pRSET/EmGFP (Novagen) and the primers EmGFP_inf_f and EmGFP_inf_r. The amplicons were inserted into the XhoI and EcoRI sites of pBBR111 to create pBBR_gfp. For infusion cloning, pBBR111 was linearized using PCR with the primers pBBR111_f_inf and pBBR111_r_inf. To construct pBBR_mamC-gfp, the MamC-GFP fusion expression vector containing the DNA fragment encoding *mamC* with the artificial C-terminal linker sequence LVPRGS was synthesized and inserted between the KpnI site and the start codon of *gfp* in pBBR_gfp. The *mamC* fragment was PCR amplified using the primers mamC_inf_f and mamC_inf_r and inserted into the linearized pBBR_gfp plasmid by using primers pBBR111_f and pBBR_gfp_r to generate pBBR_mamC-gfp. AMB-1 genomic DNA served as the template to amplify *mamI* with the primers mamI_inf_f and mamI_inf_r. We exchanged the *mamI* and *mamC* sequences in pBBR_mamC-gfp to generate pBBR_mamI-gfp. The *mamI* fragment was cloned into linearized pBBR_mamC-gfp by using the primers pBBR111_f and pBBR_gfp_linker_r. The coexpression vector pBBR_mamC-gfp/mamK, for MamC-GFP and MamK coexpression, was constructed for complementation experiments. We used PCR to insert the oligonucleotide 5′-AGGAGGACTCGAG-3′, encoding a ribosome binding site and an XhoI site, between the *mamC-gfp* and *mamK* sequences. To generate pBBR_mamC-gfp/mamK, *mamK* was PCR amplified from AMB-1 genomic DNA using the primers mamK_rbs_f and mamK_inf_r, and we used PCR to clone the amplicon into linearized pBBR_mamC-gfp with the primers pBBR_mamC-gfp/mamK_f and pBBR_mamC-gfp/mamK_r. We used inverse PCR (PrimeSTAR mutagenesis basal kit [TaKaRa Bio]) to generate pBBR_mamC-gfp/mamK^E143A^ and pBBR_mamC-gfp/mamK^D161A^ from pBBR_mamC-gfp/mamK with the following primers: for mamK^E143A^, D143A_f and D143A_r; for mamK^D161A^, D161A_f and D161A_r. These plasmids were used to transform AMB-1 cells by conjugation with *E. coli* WM3064 as described elsewhere (28).

### Imaging setup.

A culture of exponentially growing AMB-1 (30 ml) cells was centrifuged at 8,000 × *g* for 10 min at 25°C. After removal of the supernatant, the cell pellet was suspended in 5 ml of fresh MS-1 medium. We used round coverslips (25-mm diameter, 0.12 to 0.17 mm thick; Matsunami) as the imaging support. The coverslip was coated with poly-l-lysine, and 200 µl of the cell suspension was added to an Attofluor cell chamber (Thermo Fisher Scientific). The top of the chamber was covered with another coverslip to prevent drying, and then the chamber was incubated for 1 h at 28°C in the dark to allow the cells to attach to the surface of the coverslip. Next, we removed the top coverslip and placed a 15- by 15-mm, 5-mm-thick gellan gum pad (containing 0.55% gellan gum and 0.08 mM MgCl_2_ in MS-1 liquid medium) on the top of the bottom coverslip to sandwich the cells against the bottom coverslip during time-lapse imaging. The chamber was filled with fresh MS-1 liquid medium, and the top coverslip was replaced in the chamber to allow adequate microaerobic conditions to support the growth of *M. magneticum* AMB-1. During imaging, IPTG was not added to the medium, to prevent overproduction of GFP-fusion proteins.

### HILO microscopy.

Bacteria were imaged using a total internal reflection fluorescence (TIRF) microscopy-based system with an inverted microscope (Nikon Ti-E) equipped with a 100× CFI Apo TIRF objective lens (Nikon) and a 1.5× C-mount adapter (Nikon). A 488-nm laser (Sapphire; Coherent) was used to illuminate the sample at an inclined angle, which is slightly steeper than the critical angle required for total reflection in order to illuminate an entire bacterial cell. The angle of the laser beam was adjusted manually to optimize the signal-to-noise ratio. Images were acquired using a high-sensitivity electron-multiplying charge-coupled-device camera (iXon3; Andor, DU897E-CS0) with EM and preamplifier gains of 296 and 2.4×, respectively. The *Z*-position was adjusted to the best focus and was maintained by using a Perfect Focus System (Nikon) during time-lapse imaging. Time-lapse movies were taken on at least three different days using different cultures for each strain. The exposure times for GFP and bright-field images were 300 ms and 100 ms, respectively, at 1-min intervals, and the samples were illuminated only during exposure.

### Image processing.

Images were processed using the NIS Elements AR (Nikon) and ImageJ software. The only alterations to the time-lapse images were contrast adjustments using the brightness/contrast “auto” command of ImageJ. The NIS Elements AR “rotate” command was used to rotate cells into a uniform vertical orientation for kymograph analysis. Kymographs were generated using the NIS Elements AR command “show slice view” in maximum projection. The time-lapse still images were colored and merged using the ImageJ plug-in “color footprint rainbow.”

### Preparation of subcellular fractions.

Membrane, soluble, and magnetosome fractions were prepared as previously described (29). We determined the protein contents of each fraction. The membrane, soluble, and magnetosome fractions contained ~77%, ~23%, and 0.1% of the total amount of cellular protein, respectively.

### Immunochemical analysis.

Immunoblotting analysis was performed as previously described (30). Anti-GFP antibody (MBL) was diluted to 1:10,000. Goat anti-rabbit IgG conjugated to horseradish peroxidase (GE Healthcare) was diluted 1:10,000 using the Pierce Western Blotting Substrate Plus. The chemiluminescence data were collected using a luminescent image analyzer (LAS 3000; Fujifilm), and the band intensities were quantified using Multi Gauge software v.2.2 (Fujifilm). Immunofluorescence microscopy was performed as previously described (9). The anti-GFP antibody was diluted to 1:100, and an Alexa Fluor 488-conjugated goat anti-rabbit IgG antibody (Thermo Fisher Scientific) was diluted to 1:1,500.

### Physical and chemical measurements.

Protein concentrations were determined using a bicinchoninic acid protein assay kit (Thermo Fisher Scientific). SDS-PAGE was performed according to the method of Laemmli (31). Measurements of magnetic response (*C*_mag_) were performed following the method of Schüler et al. (32).

### Calculation of the diffusion coefficients of MamC-GFP foci.

The diffusion coefficients of 33 randomly selected foci in 10 Δ*mamK* cells were calculated. The center positions of the cell fluorescent foci in each still image from the live-cell time-lapse movie were determined automatically using the “analyze particles” command of ImageJ. A time-averaged mean square displacement (MSD) of the MamC-GFP foci was calculated from the trajectory of the center positions from 20 to 100 frames. The Einstein relation was used to calculate the diffusion coefficient: 4*D*Δ*t* = 〈δ^2^(Δ*t*)〉, where *D* is the diffusion coefficient of the MamC-GFP focus, Δ*t* is the time lag, 〈 δ^2^(Δ*t*) 〉 is the time-averaged MSD of the MamC-GFP focus, and 〈…〉 is the time average. We calculated 〈 δ^2^(Δ*t*) 〉 in the 1- to 6-min time-lag range and made a plot of the time-averaged MSD versus the time lag. To remove any artifacts caused by image drift, the diffusion coefficient was calculated using this plot whose slope corresponds to 4*D*.

### Calculating the theoretical value of the diffusion coefficient of a simple-diffusing magnetosome.

The theoretical value of the diffusion coefficient of a simple-diffusing magnetosome was calculated based on the formula for the relationship between the diffusion coefficients and the sizes of simple-diffusing macromolecules in *E. coli* cytoplasm (19). We considered a magnetosome chain, composed of *n* magnetosomes, as a filled sphere with a volume *n* times greater than that of a single magnetosome. The hydrodynamic radius of a magnetosome chain, *r*_p_, was defined as shown in equation 1:

(1) $$r_{p}=r_{mag}\sqrt[{3}]{n}$$

where *r*_mag_ is the hydrodynamic radius of a single magnetosome (*r*_mag_ = 25 nm). The diffusion coefficient of a magnetosome chain in the cytoplasm (*D*_cyto_) was calculated using equation 2 (19):

(2) $$\ln\left(\frac{D_{0}}{D_{cyto}}\right)={\left(\frac{\xi^{2}}{R_{h}{}^{2}}+\frac{\xi^{2}}{r_{p}{}^{2}}\right)}^{-\alpha /2}$$

where *D*_0_ is the diffusion coefficient of a magnetosome chain in water at room temperature (25°C). It is calculated using the Stokes-Sutherland-Einstein equation, *D*_0_ = kT/6πη_0_*r*_p_, where k is the Boltzmann constant, T is the absolute temperature, and η_0_ is the viscosity of water at 25°C (η_0_ = 8.9 × 10^−4^ Pa s). The fitting parameters for the cytoplasm of *E. coli* are ξ (average displacement between surfaces of proteins), *R*_*h*_ (the average hydrodynamic radius of the biggest crowders), and α (a constant on the order of 1), which were determined in a previous study (19) as follows: ξ = 0.51 nm, *R*_*h*_ = 42 nm, and α = 0.53.
