# Supplementary material for: Tethered Magnets Are the Key to Magnetotaxis: Direct Observations of Magnetospirillum magneticum AMB-1 Show that MamK Distributes Magnetosome Organelles Equally to Daughter Cells
Source: mBio. 2017 Aug 8;8(4):e00679-17. doi: 10.1128/mBio.00679-17 (PMC5550748; doi:10.1128/mBio.00679-17)
Supplement: FIG S1 [file mbo004173411sf1.pdf]

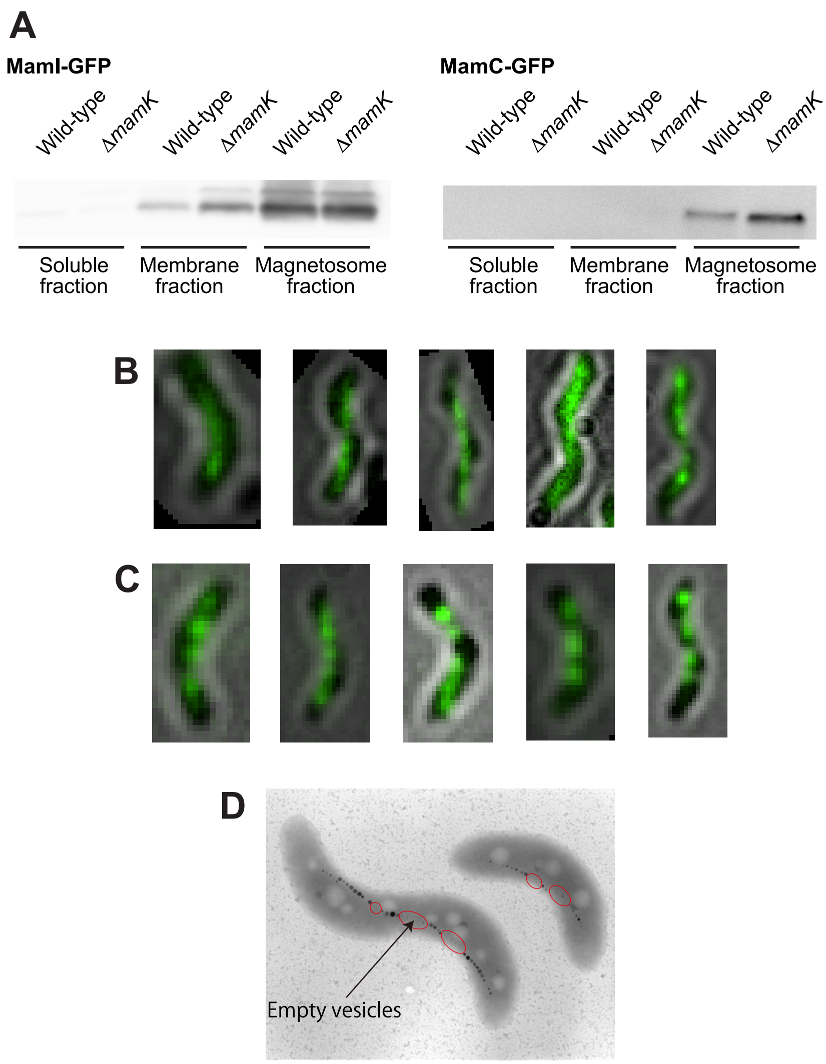

E

	Subcellular fractions		
	Soluble	Membrane	Magnetosome
Ratio of immunoblotting band intensities[a]			
MamI-GFP (N = 4)	0.95	13	100
MamC-GFP (N = 5)	0.072	0.38	100
Protein contents[b]	77%	23%	0.1%
Caliculated GFP-labeled protein contents			
MamI-GFP	19%	78%	3%
MamC-GFP	23%	36%	41%

[a] Means of band intensities in magnetosome fractions were set as 100.
[b] Percentage of total amount of cellular protein.

FIG S1 (A) Localizations of MamI-GFP and MamC-GFP in wild-type and Δ*mamK M. magneticum* AMB-1. Immunoblotting with anti-GFP antibody of proteins (10 µg/lane) extracted from the soluble, membrane, and magnetosome fractions. Both GFP-fused MamI and MamC were predominantly located in the magnetosome fractions. (B and C) Subcellular localizations of MamI-GFP and MamC-GFP. Merged GFP and bright field images of (B) MamI-GFP and (C) MamC-GFP expressed cells. (D) Transmission electron microscope image of *M. magneticum* AMB-1. The red circles show the positions of empty vesicles. (E) Summary table of MamI-GFP and MamC-GFP localizations estimated from the immunoblotting. Band intensities were measured in immunoblots of the soluble, membrane, and magnetosome fractions (5 µg protein/lane) prepared from wild-type cells. Contents of MamI-GFP and MamC-GFP in each fraction were calculated using the ratio of immunoblotting band intensities and the percentages of protein contents of each subcellular fraction.
