# Supplementary material for: Tethered Magnets Are the Key to Magnetotaxis: Direct Observations of Magnetospirillum magneticum AMB-1 Show that MamK Distributes Magnetosome Organelles Equally to Daughter Cells
Source: mBio. 2017 Aug 8;8(4):e00679-17. doi: 10.1128/mBio.00679-17 (PMC5550748; doi:10.1128/mBio.00679-17)
Supplement: TABLE S1 [file mbo004173411st1.doc]

Table S1. Phenotypes of stains

| Strains | C_mag_*^a^* | Average doubling time during live-cell imaging (n=10)*^b^* |
| --- | --- | --- |
| AMB-1 wild-type | 0.98 | Not determined |
| AMB-1 (MamC-GFP) | 0.85 | 9.6 |
| AMB-1 (MamI-GFP) | 1.31 | 5.5 |
| Δ*mamK* (MamC-GFP) | 0.88 | 7.0 |
| Δ*mamK* (MamI-GFP) | 0.83 | 10.5 |
| Δ*mamK* (MamC-GFP/MamK^WT^) | 1.02 | 8.7 |
| Δ*mamK* (MamC-GFP/MamK^E143A^) | 1.02 | 8.2 |
| Δ*mamK* (MamC-GFP/MamK^D161A^) | 1.03 | 7.1 |

*^a^* C_mag_ showed cellular magnetic response measured using light scatterring as described in Schüler et al (32).

*^b^* The doubling times were directly measured as times between cell division events using the time-lapse movies.
