# Supplementary material for: Tethered Magnets Are the Key to Magnetotaxis: Direct Observations of Magnetospirillum magneticum AMB-1 Show that MamK Distributes Magnetosome Organelles Equally to Daughter Cells
Source: mBio. 2017 Aug 8;8(4):e00679-17. doi: 10.1128/mBio.00679-17 (PMC5550748; doi:10.1128/mBio.00679-17)
Supplement: FIG S2 [file mbo004173411sf2.pdf]

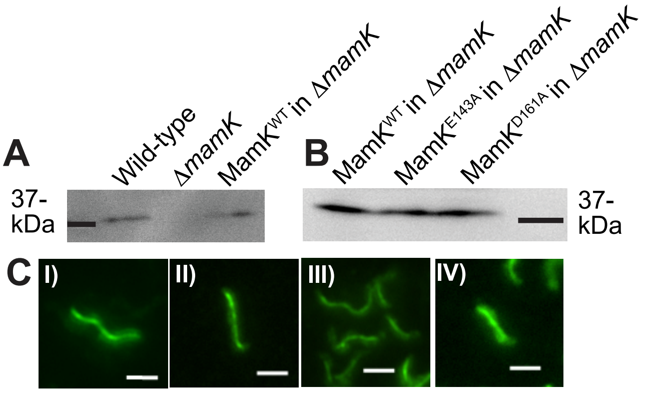

FIG S2 Expression of MamK^{WT}, MamK^{E143A} and MamK^{D161A} in Δ*mamK* cells. (A) Immunoblotting with anti-MamK antibody of cell free extracts (10 µg protein/lane) from wild-type, Δ*mamK*, and the MamK-complemented strains. The MamK content of the complemented strain was similar to that of the wild-type starain. (B) Immunoblotting with the anti-MamK antibody of cell free extracts (10 µg protein/lane) from the MamK-complement strain, and MamK^{E143A} and MamK^{D161A} expressed Δ*mamK* cells. (C) Immunofluorescence images of I) wild-type, II) MamK-complement strain, III) MamK^{E143A} and IV) MamK^{D161A} expressed Δ*mamK* cells with the anti-MamK antibody. The MamK ATPase mutants, MamK^{E143A} and MamK^{D161A}, showed linear filamentous localizations similarly to MamK^{WT}.
