# Supplementary material for: Tethered Magnets Are the Key to Magnetotaxis: Direct Observations of Magnetospirillum magneticum AMB-1 Show that MamK Distributes Magnetosome Organelles Equally to Daughter Cells
Source: mBio. 2017 Aug 8;8(4):e00679-17. doi: 10.1128/mBio.00679-17 (PMC5550748; doi:10.1128/mBio.00679-17)
Supplement: TABLE S2 [file mbo004173411st2.doc]

Table S2. Bacterial stains and plasmids used in this study

| Strains or plasmids | Relevant characteristics | References or sources |
| --- | --- | --- |
| ***M. magneticum* AMB-1** |  |  |
| AMB-1 | Wild type (ATCC700264) | (1) |
| Δ*mamK* | *mamK* (non-polar deletion mutant) | (2) |
|  |  |  |
| ***E. coli*** |  |  |
| XL-1 blue MRF’ | Δ(*mcr*A)183 Δ(*mcr*CB-*hsd*SMR-*mrr*)173 *end*A1 *sup*E44 *thi*-1 *rec*A1 *gry*A96 *rel*A1 *lac* [F’,*pro*AB,laqI^q^Z ΔM15, Tn10(Tet^R^)] | Stratagene |
| WM3064 | *thrB1*004 *pro thi rpsL hsdS lacZ*Δ*M15* RP4-1360 Δ(*araBAD)567* Δ*dapA*1341::[*erm pir*] | (3) |
|  |  |  |
|  |  |  |
| **Plasmids** |  |  |
| pBBR111 | Protein expression vector for *M. magneticum* AMB-1, tac promoter, Mob*,* Km^R^ | (4) |
| pRSET/EmGFP | Source of *gfp* gene | Novagen |
| pBBR_gfp | pBBR111 carrying *gfp* gene | This study |
| pBBR_mamC-gfp | pBBR111 carrying *mamC-gfp* | This study |
| pBBR_mamI- gfp | pBBR111 carrying *mamI-gfp* | This study |
| pBBR_mamC-gfp/mamK | pBBR111 carrying *mamC-gfp and mamK* | This study |
| pBBR_mamC-gfp/mamK^E143A^ | pBBR111 carrying *mamC-gfp and mamK^E143A^* | This study |
| pBBR_mamC-gfp/mamK^D161A^ | pBBR111 carrying *mamC-gfp and mamK^D161A^* | This study |

**Supplemental references**

1. **Matsunaga T, Sakaguchi T, Tadakoro F.** 1991. Magnetite formation by a magnetic bacterium capable of growing aerobically. Appl Microbiol Biotechnol 35:651-655.

2. **Komeili A, Li Z, Newman DK, Jensen GJ.** 2006. Magnetosomes are cell membrane invaginations organized by the actin-like protein MamK. Science 311:242-245.

3. **Komeili A, Vali H, Beveridge TJ, Newman DK. 2004.** Magnetosome vesicles are present before magnetite formation, and MamA is required for their activation. Proc Natl Acad Sci U S A 101:3839-3844.

4. **Philippe N, Wu LF.** 2010. An MCP-like protein interacts with the MamK cytoskeleton and is involved in magnetotaxis in Magnetospirillum magneticum AMB-1. J Mol Biol 400:309-322.
