# Supplementary material for: Tethered Magnets Are the Key to Magnetotaxis: Direct Observations of Magnetospirillum magneticum AMB-1 Show that MamK Distributes Magnetosome Organelles Equally to Daughter Cells
Source: mBio. 2017 Aug 8;8(4):e00679-17. doi: 10.1128/mBio.00679-17 (PMC5550748; doi:10.1128/mBio.00679-17)
Supplement: TABLE S3 [file mbo004173411st3.doc]

Table S3. Primers used in this study

| Primer names | Sequences (5´-3´) |
| --- | --- |
| EmGFP_inf_f | ACAGGAGGACTCGAGATGGTGAGCAAGGGCGAGGAG |
| EmGFP_inf_r | GGGCTGCAGGAATTCTCACTTGTACAGCTCGTCCATGC |
| pBBR111_f_inf | CTCGAGTCCTCCTGTTTCCTGTG |
| pBBR111_r_inf | GAATTCCTGCAGCCCGGGGGATCCAC |
| pBBR_gfp_r | ATGGTGAGCAAGGGCGAGGAGC |
| mamC_inf_f | ACAGGAGGACTCGAGATGCCCTTTCACCTTGCCCCC |
| mamC_inf_r | GCCCTTGCTCACCATGGAACCGCGTGGCACCAGGGC |
| mamI_inf_f | ACAGGAGGACTCGAGATGCCAAGCGTGATTTTCGGACTGC |
| mamI_inf_r | ACCGCGTGGCACCAGACCATCGATGTCAGGGTCTGAAGCG |
| pBBR_gfp_linker_r | CTGGTGCCACGCGGTTCCATGGTG |
| mamK_rbs_f | GAAGGAGGACTCGAGATGAGTGAAGGTGAAGGCCA |
| mamK_inf_r | GGGCTGCAGGAATTCTCACGAGCCGGAGACGTCTC |
| pBBR_mamC-gfp/mamK_f | CTCGAGTCCTCCTTCACTTGTACAGCTCGTCCA |
| pBBR_mamC-gfp/mamK_r | CGTGAGAATTCCTGCAGCCCGGGGGAT |
| D143A_f | GTGTCCGCCCCGTTCATGGTCGGCTAC |
| D143A_r | GAACGGGGCGGACACCACTAGGGCGG |
| D161A_f | TCGTCGCCATCGGCGCCGGAACCACG |
| D161A_r | CGCCGATGGCGACGATGATGGTGTTGA |
